# CancerNet: a database for decoding multilevel molecular interactions across diverse cancer types

**DOI:** 10.1038/oncsis.2015.40

**Published:** 2015-12-21

**Authors:** X Meng, J Wang, C Yuan, X Li, Y Zhou, R Hofestädt, M Chen

**Affiliations:** 1 Department of Bioinformatics, College of Life Sciences, Zhejiang University, Hangzhou, China; 2 James D Watson Institute of Genome Sciences/Institute of Bioinformatics, Zhejiang University, Hangzhou, China; 3 Department of Bioinformatics and Medical Informatics, Faculty of Technology, Bielefeld University, Bielefeld, Germany

## Abstract

Protein–protein interactions (PPIs) and microRNA (miRNA)–target interactions are important for deciphering the mechanisms of tumorigenesis. However, current PPI databases do not support cancer-specific analysis. Also, no available databases can be used to retrieve cancer-associated miRNA–target interactions. As the pathogenesis of human cancers is affected by several miRNAs rather than a single miRNA, it is needed to uncover miRNA synergism in a systems level. Here for each cancer type, we constructed a miRNA–miRNA functionally synergistic network based on the functions of miRNA targets and their topological features in that cancer PPI network. And for the first time, we report the cancer-specific database CancerNet (http://bis.zju.edu.cn/CancerNet), which contains information about PPIs, miRNA–target interactions and functionally synergistic miRNA–miRNA pairs across 33 human cancer types. In addition, PPI information across 33 main normal tissues and cell types are included. Flexible query methods are allowed to retrieve cancer molecular interactions. Network viewer can be used to visualize interactions that users are interested in. Enrichment analysis tool was designed to detect significantly overrepresented Gene Ontology categories of miRNA targets. Thus, CancerNet serves as a comprehensive platform for assessing the roles of proteins and miRNAs, as well as their interactions across human cancers.

## Introduction

Cancer is a complex disease characterized by a large number of molecular interaction alterations.^1^ Distinguishing the bona fide drivers of cancer phenotypes has proven to be a daunting task, which is further exacerbated by the complexity of elucidating how such drivers interact synergistically to elicit cancer phenotypes. Thus, a systems biology approach, the analysis of multilevel molecular interactions, is required to understand the pathogenesis of human cancers.^2, 3^

Protein–protein interaction (PPI) networks provide a global picture of cellular functions and biological processes,^4^ the dysfunction of some interactions causes many diseases, including cancer. Therefore, the use of PPI networks has become one of the major and powerful approaches to elucidate the molecular mechanisms underlying the complex diseases on the system level.^5, 6^ Recently, Barshir *et al.*^7^ developed the TissueNet including tissue-specific and tissue-wide PPIs across 16 human normal tissues. Veres *et al.*^8^ constructed a cellular compartment-specific database for PPI network analysis. However, these databases do not provide cancer-related PPIs. Thus, a disease analysis is not allowed. However, it is valuable to construct a more comprehensive database to store context-specific PPIs across varieties of cancer and normal tissues. This is especially the case because of the tremendous increase in human protein interaction data, as well as expression data in both cancer and normal tissues produced by large-scale projects, such as The Cancer Genome Atlas^9^ and NIH Roadmap Epigenomics Mapping Project.^10^

MicroRNAs (miRNAs), as master gene regulators, have crucial roles in disease-associated processes.^11, 12^ A single miRNA is capable of regulating >200 mRNAs, and a single mRNA may be regulated by multiple miRNAs,^13^ but a substantial fraction of these interactions may depend on the cell type and/or context^14^ and function in specific tissues.^15^ Therefore, identification of context-dependent miRNA–target interactions is quite important to study the pathogenesis of complex diseases.^16, 17^ Mammalian miRNAs can influence gene expression not only by inhibiting protein translation but also by decreasing target mRNA levels that causing a negative correlation between the expression levels of miRNAs and their target mRNAs.^18, 19, 20^ As a result, the inverse expression relationship between miRNAs and mRNAs are frequently used to predict miRNA targets.^21, 22, 23^ MiRNA expression broadly contributes to tissue specificity of mRNA expression in many human tissues.^24^ Therefore, by integrating gene expression data across a variety of cancers into the global miRNA–target regulatory network, cancer-specific and cancer-wide miRNA–target interactions can be inferred.

Multiple miRNAs can synergistically regulate one or more pathways by targeting common or functionally similar targets.^25^ In addition, the synergism may change in different diseases. Therefore, detecting the miRNA synergism across diverse cancer types is essential for uncovering pathogenesis of human cancers in a global sense. Several methods have been developed to discover miRNA synergism. Xu *et al.*^25^ constructed a miRNA–miRNA functionally synergistic network via co-regulating functional modules while Li *et al.*^26^ detected synergistic miRNA regulatory modules by overlapping neighborhood expansion. These studies have demonstrated the importance of miRNA synergism. However, the researchers only focused on several cancer types. Therefore, a pattern analysis across diverse cancer types is unavailable. Here we present a novel approach to identify miRNA synergism across human cancer types. For each cancer, functional similarity between miRNAs was estimated by measuring the similarity of their associated target genes, and the proximity of target genes in the PPI network was further detected. Our methods considered both the biological functions of miRNA targets and their topological structure in the PPI network. A systematic insight into the nature and scale of the potential synergistic interactions is essential for the study of complex diseases.

In this study, we developed a new database CancerNet, which is a cancer-specific database that provides multilevel molecular interactions across diverse cancer types. Users can retrieve cancer-wide or cancer-specific PPIs, miRNA–target interactions and functionally synergistic miRNAs using multiple query methods. CancerNet also provides enrichment analysis for miRNA targets and a network visualization tool. Above all, CancerNet serves as an important data resource that can help researchers to understand the regulatory mechanisms or interaction patterns between different molecules across diverse cancer types.

## Results

### Overview of CancerNet

CancerNet aims to provide cancer-specific molecular interaction networks across multiple cancer types. Currently, 33 human cancer types are included. The interactions contain PPIs, miRNA–target interactions and miRNA–miRNA synergistic interactions. Experimentally detected PPIs were assembled from five major PPI databases and miRNA–target interactions were considered as the combination of the predicted targets from six algorithms and two experimentally validated data sets, amounting to 185 589 PPIs and 3 249 385 miRNA–target interactions, respectively. Synergistic miRNA pairs were predicted according to the functions of target genes as well as their proximity in the PPI network. By integrating expression data in different cancer samples and information from Gene Ontology (GO) annotations, cancer-wide and cancer-specific molecular interactions were identified. CancerNet offers a unique platform for assessing the roles of human proteins and miRNAs, as well as their interactions across human cancers.

### Query and result description

#### Query

A flexible and user-friendly query method is provided by CancerNet, users can query it using one molecule to retrieve its interaction partners per cancer, or using a pair of interacting molecules to retrieve the cancer types that the interaction appears in. For each molecule, two types of identifier can be used. In case of a miRNA, the name or accession number in miRBase can be used, while in case of a gene, the official symbol or Entrez ID can be used.

#### Result list

CancerNet output data provide lists of interactions and detail information about the interactions. Expression levels and functional similarity score are provided for the PPI and miRNA–miRNA result list, respectively. And for the miRNA–target result list, the Pearson correlation coefficient and *P-*value are offered. In addition, users can check the cancer specificity of each interaction from the last column of the result list. Specifically, for each functionally similar miRNA pair, users can further explore the enriched GO terms of their targets.

#### Visualization

One molecule may interact with multiple molecules in the same context. Therefore, these interactions form a complex regulatory network. To visualize these networks, a graphical network viewer based on the Cytoscape Web program^27^ was developed. Edges (interactions) are colored distinctly according to their associated cancer types. In addition, users can use a special toolkit of ‘My PPI/miRNA-Target/miRNA-miRNA Network', which contains previously stored interactions of interest. Consequently, this toolkit supports users in discovering specific biological network formed by molecular interactions that involved in regulation of the interrelated processes.

#### Enrichment analysis

Functional enrichment analysis of miRNA target genes are widely used to detect the miRNA functions. A ‘GO Enrichment Analysis' module has been developed for miRNA–target list based on GO annotation. The *χ*^2^-test and Fisher's exact test were used to evaluate the significance of enrichment for GO terms. This module facilitates users to explore the biological functions of target gene set that they are interested in.

### Biological applications

Multilevel molecular interactions are involved in tumorigenesis. Therefore, deciphering these interactions is essential for cancer research. CancerNet enables researchers to comprehensively view the interactions formed by the molecules of interest across diverse cancer types. Combination treatment with these interactions has an important role in cancer research.^28^ In this section, we will show how to use CancerNet to find useful information.

MiR-1 and miR-133a have been frequently found to be co-downregulated in several types of cancer.^29, 30, 31, 32, 33, 34^ Furthermore, overexpression of both of them inhibited cancer cell proliferation and induced cancer cell apoptosis.^32, 35, 36^ Therefore, there must be a functional link between miR-1 and miR-133a. Next, this miRNA pair will be further discussed using our system.

**Figure 1:**
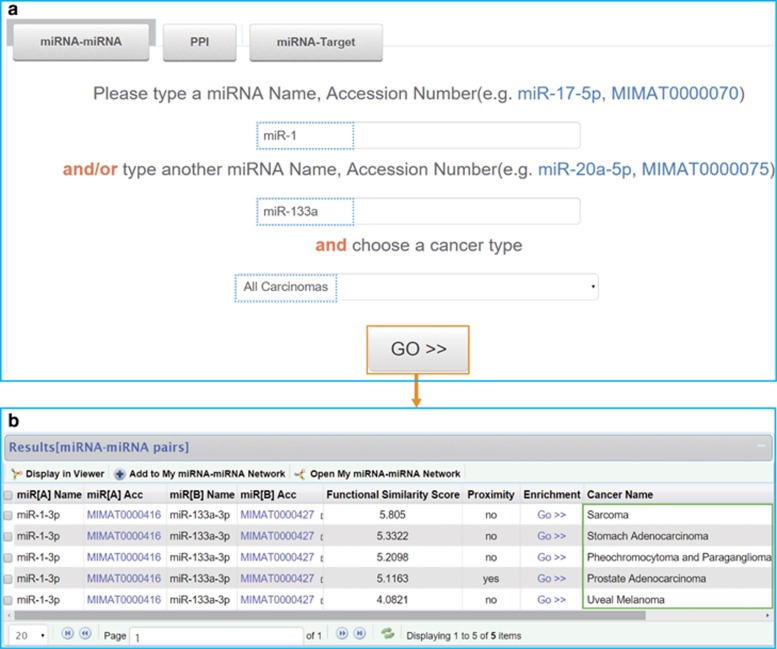
Synergism between miR-1 and miR-133a. (**a**) Screenshots of the search section. The search engine includes three parts: miRNA–miRNA; PPI; and miRNA–target, allowing users to query functionally synergistic miRNA pairs, PPIs and miRNA–target interactions, respectively, across diverse cancer types. As an example, the miR-1 and miR-133a were queried. (**b**) Result list of miR-1 and miR-133a. Synergism between miR-1 and miR-133a exists in five types of cancers, shown in green box.

First, we are curious about in which cancer types this miRNA pair exists, so miR-1 and miR-133a were queried simultaneously in CancerNet (Figure 1). From the result page, we could find that miR-1 and miR-133a are functionally synergistic in five cancer types: sarcoma; stomach adenocarcinoma; pheochromocytoma–paraganglioma; prostate adenocarcinoma; and uveal melanoma. Among them, it has been revealed that both miR-1 and miR-133a are related to sarcoma,^30^ stomach adenocarcinoma^37, 38^ and prostate adenocarcinoma.^39^ MiR-133b, which is from the miR-133 family (miR-133a and miR-133b), potentially regulates pathways related to pheochromocytoma–paraganglioma.^40^ In addition, expression level of both miR-1 and miR-133a altered in uveal melanoma.^41^ Accordingly, the synergism between miR-1 and miR-133a revealed by CancerNet is consistent with previous studies.

**Figure 2:**
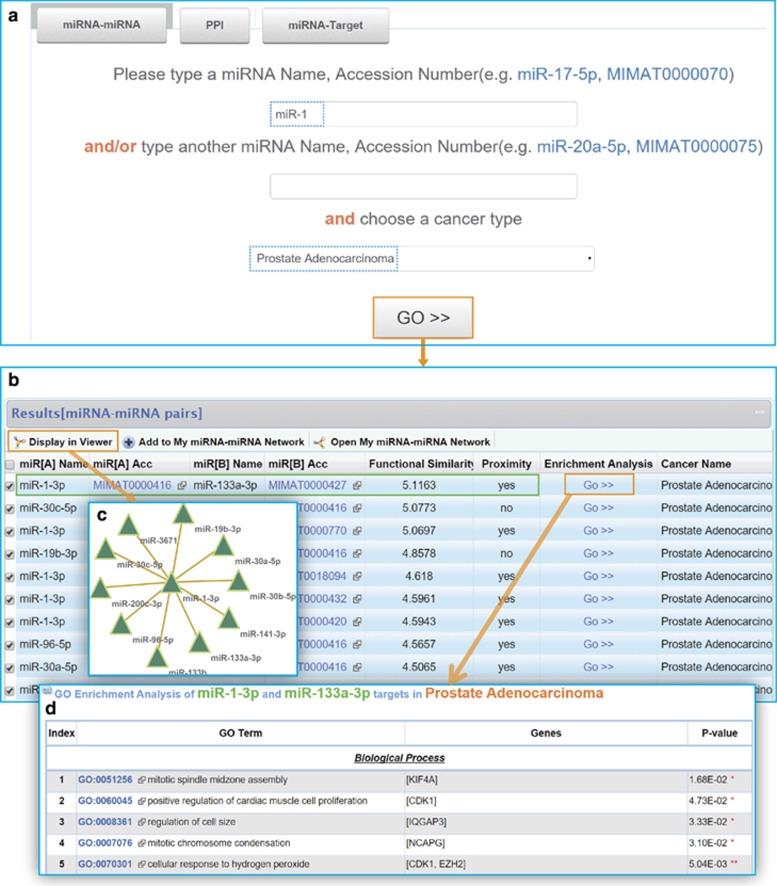
Functionally synergistic miRNA pairs formed by miR-1. (**a**) Screenshots of the search page. (**b**) Result list of miR-1. MiR-1 and miR-133a share the highest functional similarity score, shown in the green box. (**c**) Functionally synergistic miRNA–miRNA network (top 10 are displayed). (**d**) Results of functional enrichment analysis.

**Figure 3:**
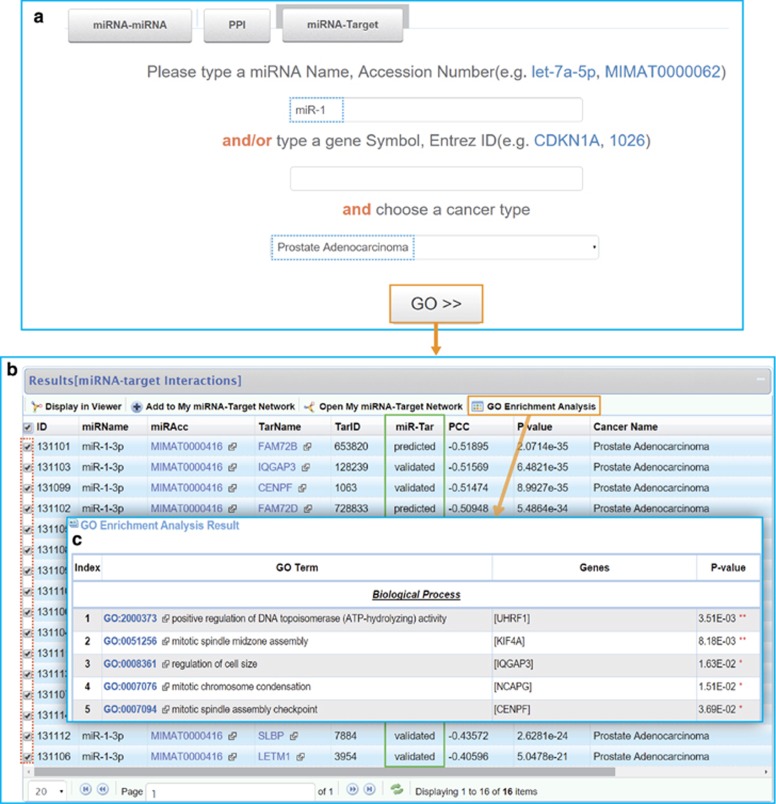
Targets of miR-1 in prostate adenocarcinoma. (**a**) Screenshots of the search page. (**b**) Result list of miR-1 targets. Most miRNA–target interactions (12/16) have been experimentally validated, shown in green box. (**c**) Results of functional enrichment analysis.

Second, we would like to further detect all the miRNAs that are functionally synergistic with miR-1 or miR-133a in a certain cancer type such as prostate adenocarcinoma. This can be easily achieved by our system. Here we only show the results of miR-1 (Figure 2). Of all the pairs formed by miR-1 in prostate adenocarcinoma, miR-133a and miR-1 share the highest functional similarity score that means their functions are closely linked. Besides, their targets are proximate in the PPI network. Then we want to find out the pathways that their targets are associated with. To realize this, a functional enrichment analysis tool has been embedded in the result page that allows users to discover the significant GO terms for the targets of each miRNA pair. The targets of miR-1 and miR-133a are enriched in cancer-related GO biological processes such as mitotic cell cycle (*P*=1.13E-08), cell division (*P*=2.54E-08) and mitotic nuclear division (*P*=9.74E-08). Our system also provides a graphic viewer to visualize interactions that users are interest in. In addition, the targets of miR-1 or miR-133a in prostate adenocarcinoma could be further queried (Figure 3). For miR-1, most miRNA–target interactions are experimentally validated (12/16). Similarly, we can select the targets of interest and perform a functional enrichment analysis.

**Figure 4:**
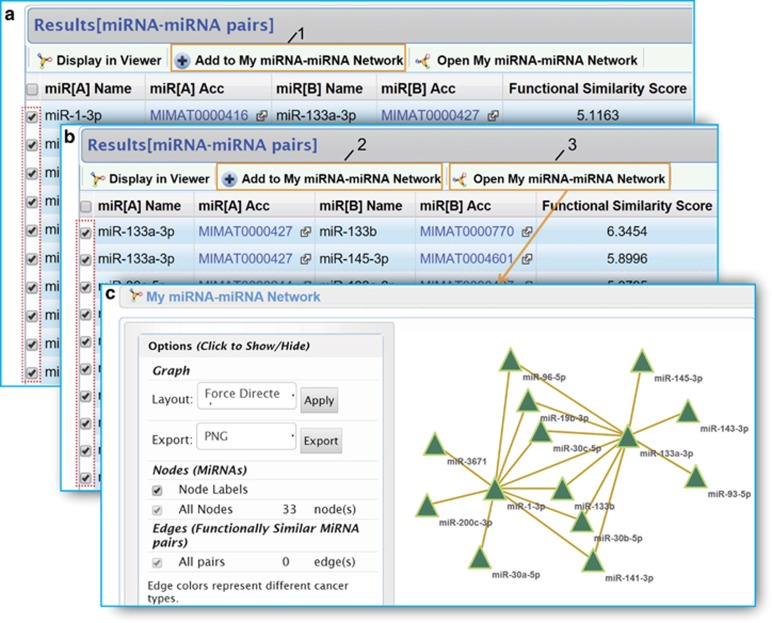
Network visualization of the common partners that are synergistic with miR-1 and miR-133a in prostate adenocarcinoma. (**a**) Result list of miR-1 partners. Top 10 items are added to ‘My miRNA-miRNA Network'. (**b**) Result list of miR-133a partners. Top 10 items are added to ‘My miRNA-miRNA Network'. (**c**) ‘My miRNA-miRNA Network'.

Finally, CancerNet provides a way to study functionally synergistic miRNAs on the system level. For example, the common partners that are synergistic with miR-1 and miR-133a in prostate adenocarcinoma can be visualized by using ‘My miRNA-miRNA Network' module (Figure 4). The six common partners (miR-19b, miR-30b, miR-30c, miR-96, miR-133b and miR-141) are all related with prostate cancer. MiR-19b promotes prostate cell proliferation with regulation of PTEN and its downstream signals.^42^ Overexpression of miR-30 in prostate cancer cells suppresses epithelial-mesenchymal transition (EMT) phenotypes and inhibits cell migration and invasion.^43^ Upregulation of miR-96 enhances cellular proliferation of prostate cancer cells through FOXO1.^44^ Overexpression of miR-133b in LNCaP cells boosts cell proliferation and cell cycle progression, but inhibits apoptosis.^45^ MiR-141 modulates androgen receptor transcriptional activity in human prostate cancer cells through targeting the small heterodimer partner protein.^46^ Together with miR-1 and miR-133a, these miRNAs form a functionally synergistic clique in prostate cancer and jointly function in tumorigenesis. Therefore, a combinatorial study of these synergistic miRNAs is important for understanding the mechanisms of tumorigenesis and finding bona fide biomarkers for cancer diagnosis and prognosis.

From our system, we could find out that co-expression of miR-1 and miR-133a is meaningful. They synergistically regulate functionally related genes or pathways in several cancer types. Understanding the synergism between them is quite useful to elucidate the roles they have in tumorgenesis.

## Discussion

Knowledge of molecular interactions is quite useful for discovering the functions of molecules and the processes they are involved in. However, there is little systematic insight into the nature and scale of the potential interactions in human cancers. Although human PPIs are accessible through several public databases, these databases do not specify the human cancer types in which these PPIs take place. In addition, there are no databases can be used to retrieve cancer-specific miRNA–target interactions as well as functionally synergistic miRNA pairs. However, by integrating high-throughput sequencing data, we can discover these interactions across diverse cancer types. To discover the miRNA synergism, we measured the functional similarity of targets in the miRNA–target network and the target proximity in the PPI network in each cancer type. These results could help researchers to elucidate the roles miRNAs have in tumorigenesis from a system-level perspective.

In summary, CancerNet provides a unique data resource for the analysis of PPI networks, miRNA–target regulatory networks and functionally synergistic miRNA–miRNA networks across diverse cancer types. Studying these cancer-specific interactions will help researchers to detect the interaction patterns across human cancers, discover the true cancer-related regulatory modules and understand the mechanisms of tumorigenesis. In addition, it allows researchers to quickly identify poorly annotated miRNAs or protein-coding genes interacted with molecules, which have already been well studied. Therefore, our system provides guidance on studying the functions of these molecules, especially their roles in human cancers, and has significant implications for miRNA combination therapy of human cancers.

## Materials and Methods

### Expression data sources and processing

Level 3 miRNA-Seq and RNA-Seq data were obtained from the The Cancer Genome Atlas data portal (https://tcga-data.nci.nih.gov/tcga/tcgaHome2.jsp), including 33 cancer types, 8296 tumor samples in all. Reads per million miRNA mapped values were used to represent miRNA expression levels. For RNA-Seq data, upper quartile normalized gene counts were calculated using the RSEM algorithm,^47^ RSEM's gene level expression estimates were multiplied by 1 000 000 to obtain transcript per million (TPM) estimates for each gene, and TPM estimates were transformed to log-space by taking log_2_(TPM+1). Normal tissue and cell line RNA-Seq data were downloaded from Illumina Body Map 2.0^48^ and NIH Roadmap Epigenomics Mapping Project.^10^ Raw reads were mapped to hg19 using TopHat 2.0.11^49^ and gene expression levels were quantified by Cufflinks 2.2.0.^50^

### Protein–protein interactions

We compiled experimentally identified PPIs from BioGRID,^51^ DIP,^52^ HPRD,^53^ IntAct^54^ and MINT.^55^ Only physical interactions were retained and these data are amounted to 185 589 PPIs between 16 278 human proteins. For each cancer type, genes were considered expressed if their transformed expression level was equal to or above 2 (in log_2_(TPM+1) scale) in at least 80% samples. Whereas, for each normal tissue or cell type, mean expression values were calculated and genes with at least two FPKM (fragments per kilobase of exon per million fragments mapped) were considered expressed. Then, a PPI was associated with a cancer or normal tissue if the two pair mates were both found to be expressed in that cancer or normal tissue.

### MiRNA–target interactions

MiRNA sequences were extracted from the miRBase database (release 21).^56^ MiRNA target genes were acquired from six common miRNA target-predicting programs, including DIANA-microT,^57^ miRDB,^58^ PITA,^59^ RNAhybrid,^60^ TargetScan^61^ and miRanda.^62^ In order to improve the reliability of the predicted target genes, only targets predicted by at least two of these programs were retained and further incorporated with two experimentally validated data sets from miRecords^63^ and miRTarBase.^64^ Next, the miRNA–target interactions existing in a cancer type were produced according to the inverse expression relationships between miRNAs and their target mRNAs. Thus, the Pearson correlation coefficients were calculated for each miRNA–target pair, whose threshold was set to <−0.4 and corresponding *P*-value was set to <0.01.

### MiRNA–miRNA synergism

To identify functionally synergistic miRNA pairs in a specific type of cancer, the functional similarity of targets was first calculated. Then, the proximity of targets in that cancer PPI network was measured.

The functional similarity scores were calculated for each miRNA pair using FastSemSim (http://sourceforge.net/p/fastsemsim) based on the GO biological process terms,^65^ and the threshold was set to >5. We used information content to evaluate semantic similarity of GO terms, as defined by Resnik.^66^ Here for each miRNA in a certain cancer type, only the targets existing in the miRNA–target interaction network from that cancer were used.

For each pair of miRNAs, if their targets tend to be proximate in the PPI network, we considered that these two miRNAs share common functions as closely linked nodes tend to form a function module in the PPI network.^67^ We presented a method to measure the proximity of targets in a network, which considered the proximity between two target sets as well as the local proximity of all the targets. Therefore, the proximity between one target and one target set has to be defined. Here we let ‘t' represents one target and let ‘TS' represents one target set. Then we define the proximity between t and TS, *p*(t,TS), as the sum of shortest path length from t to each member of TS, for example, TS={ts_1_, ts_2_, …ts_*k*_}. It is calculated as follows:


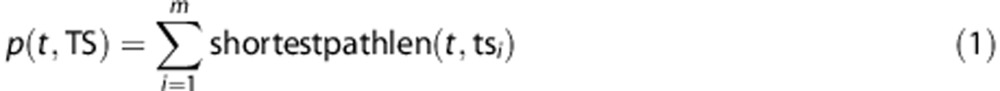


Next, if target set *A* contains *m* genes and target set *B* contains *n* genes, then the proximity between *A* and *B* is defined as


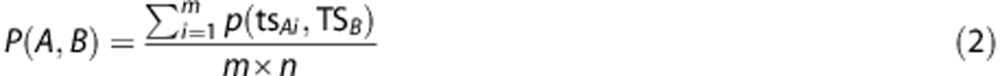


And the local proximity formed by *A* and *B* is defined as


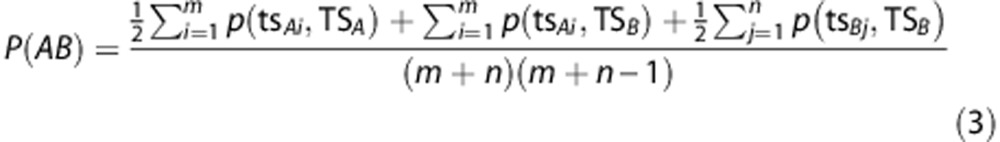


which measures the local connectivity among all the genes from *A* and *B*. Then we set the threshold for *P*(*A*,*B*) and *P*(*AB*) to <1.5 and <0.7, respectively, to determine whether two miRNAs have a synergistic relationship.
